# Answering to the domesticability of exotic options and strategies in managing Africa’s urban landscapes for sustainability beyond 2015

**DOI:** 10.1186/2193-1801-3-241

**Published:** 2014-05-09

**Authors:** Innocent EW Chirisa, Shingai T Kawadza, Elmond Bandauko

**Affiliations:** Department of Rural and Urban Planning, University of Zimbabwe, PO Box MP167, Mt Pleasant, Harare, Zimbabwe; Urban Development Corporation, 3rd Floor, Throgmorton House, 51 Samora Machel/Julius Nyerere Way, Harare, Zimbabwe

**Keywords:** Exotic, Domesticating policy, Urban management, Landscape, Corruption, Development

## Abstract

This study aims at critically assessing the land management strategies that can be instrumental in bringing sound governance to urban landscapes in Africa with the view of mapping the potential, minimum conditions for success and constraints to doing so. This study is qualitative by approach and case study based by design, assesses practices in land management from a few cities (Nairobi, Abuja, Harare, Kigali, Johannesburg and Addis Ababa). Peculiarities and differences in the practices of land management in these cities is the basis for their purposeful selection. The evaluation of the land management practices in these cities is in terms of the current realities and the possibility for the acceptability of new, exotic but deemed sustainable urban land management styles. Noted strongly in this current discourse is that Africa is a region with varied of contexts requiring a critical assessment of issues before policy strategies are implemented in terms of land tenure, land administration corruption, political will and receptivity of the so-called foreign philosophies in urban land governance. The study recommends relevant training of the land and planning experts in Africa. In addition, there is general need to balance between ‘place prosperity’ with ‘people prosperity’ as they relate to land management noting that space and capital make the difference in sustainable human habitats’ creation and management.

## Introduction

Africa is in a mess of increasing urbanisation and urban poverty. This mess has crystallised into eyesores manifesting in increasing housing informality, uncollected garbage and the rampancy of the informal sector activities in the urban landscape. The summary of it all is in the shortcomings of viable land governance options. Informal land deals, limited technological advancement to enhance management, limited and acceptable land tenure options, political exigencies impinging on human rights and sound urban management including urban planning, structural problems of the market, to name but these few are the major challenges. The urban populations are often in limbo with respect to what they can do for themselves as they are caught in between forces of market and state failures that stifle the efficient practice of land delivery. Although there are a host of strategies and options that have been applied in Asia (for example China and Singapore) and Latin America (for example Brazil, Argentina and Venezuela) with meaningful success, little remains known of how the same can be domesticated in Africa. For example, (van der Krabben 2012) has suggested innovations in land management including the acquisition of land and properties. (Acioly 2007): 6–7, on the other hand, has suggested a number of strategies. These include but not limited to titling of individual properties for example the COFOPRI experience in Peru (Program for the Formalisation of Properties), the establishment of special zones of social interest (ZEIS), the innovative national, slum networking programmes in India and transfer of development rights, land value capture to boost infrastructure development. For (Aribigbola 2008): 2, “…effective urban land control … is crucial to tackling growing land use problems such as slum formation, rising costs of land, accessibility to urban land for land housing, incompatible use, flooding, overcrowding and congestion among others for the purpose of achieving sustainable city development and ensure the safety and health of the people.” In China, according to (Kremznert 1998): 645 all “… municipal governments are required to formulate two types of urban plans that “scientifically forecast local needs”. With all this evidence of success, the question of possibilities and limitations in applicability is a major one in the context of African cities. Unless, there is testing of the ground regarding what it can or cannot withstand, Africa’s urban landscapes are likely to get deeper into problems when options are available. This study is useful in finding the appropriate strand and minimum conditions for success and progress in addressing urban land management problems.

Africa is a region with a variety of contexts (historical, geographical and socio-economic) that require a critical assessment of issues before implementation of policy strategies. The critical domains in the regions for exploration are land tenure arrangements, land administration corruption, the political will and receptivity of so-called foreign philosophies. Relevant training of land and planning experts is a key requisite in urban development practice in Africa. In addition, value generation in land and the benefits accrue to urban centres for the meaningful infrastructure development and ultimately the people – balancing place prosperity with people prosperity. The post-2015 must be a period of practical approaches to moving people out of the deep poverty associated with urbanisation. In all this, land, development, space and capital make the difference. A diagnosis on urban land management in Africa with the view of assessing the terrain so that the existing options, including those that have been successful elsewhere can be retrofitted to improve the situation in the post-2015 era is long overdue. Unless we put these to proper context, the current situation persists, Africa’s cities will be more entrenched into problems deeper and eyesores in the urban landscapes coupled with ‘bad politics’ on land will deepen the poverty situation. This present article aims at critically assessing the land management strategies that can be instrumental in bringing sound governance to urban landscapes in Africa with the view of mapping the potential, minimum conditions for success and constraints to doing so. It is organised under the following headings: strategies and options for urban land management: context and review; profiling of land management practices in selected African cities; emerging issues: sifting between expectations and realities; discussion, policy implications and recommendations; and, conclusion.

## Strategies and options for urban land management: context and review

Largely, urban land management falls within the ambit of local government. Past practice made local government subordinate and dependent on central government resources, a development that tended to cripple it as it could not harness sufficient local tax let alone address the local social, economic and environmental challenges. (Boyle *et al*. 2003): 11 argue that the incapacity of the local government to coordinate local development have paved way for weak control leading to increased informality in land use and development. (Acioly 2003): 1 defines urban management as “… a set of instruments, activities, tasks and functions that assures that a city can function.” Within the realm of urban management is urban land management of which one major conceptual problem is to equate land administration to land management (CITYNET, 1995). The dynamism in land management assumes the active participation of the citizens including the importance of future generations in the plans. Dynamic planning, public-private partnership, low-cost technologies and appropriate standards are key strategies for sustainability. In the developing countries, a number of factors often hamper sustainable urban land management. These include, among other things, lack of a comprehensive land policy taking on board all agencies, over-centralisation of land administration and urban planning, limited enabling capacity, lack of adequate financial resources at the local level, complexity in land regulations and procedures, multiple land tenure systems and lack of information (Magel and Wehrmann, 2002): 3–4. These challenges tend towards corrupt practices by bureaucrats and politicians. For (Harris 2012): 178, “… lack or weakness in important institutions is an important productivity trap and a defining aspect of informality.” The major manifestation of urban informality is in the housing sector, which is the major explanation behind slum formation in most cities and towns (Acioly, 2007): 2.

As portrayed by the Urban LandMark (2011): 3 “… a city’s urban land market is both a barometer of its success and of its problems.” From an economic viewpoint, four components define the land market. These are land, capital, development and space. The four components are interdependent and interlocking. (Acioly 2012):16 assert that, “scarcity of one component brings disequilibrium”. Apart from the structural aspects of land management in urban areas, there are also the natural processes to needing factoring in. Specifically, this has to be in light of the sustainable development agenda hence an ecosystems approach to development practice (Boyle *et al*., 2003; Heckbert et al., 2008): 4. Several tools are important to assist in this direction including, among other things applications of geographic information systems (GIS), strategic environmental assessment (SEA) and life–cycle analysis (LCA). New demands on land, like urban agriculture, are on the horizon (Cabannes 2003:1and (Karlenzig 2012):4. These measures are important as measures of adaptation and mitigation of the adverse impacts of climate change. (Mund et al. 2005):2–3 citing Sachs (2003) has argued that effective urbanisation is pillared on three distinct policy dimensions namely urban planning, urban development strategy, and urban governance. With reference to Cambodia, they observe that the urban master plan process is inefficient and decentralisation of functions at national level is not well coordinated. Table 1 by (van der Krabben 2012): 5 outlines goals and supporting tools for effective land management (See Table 1). Table 2 indicates institutions involved in land management and their specific roles with reference to Johannesburg (South Africa). Legislation is critical to ensure use orderliness and certainty in land management. Table 3 shows the essential legal instruments used in Zimbabwe.

**Table 1:** **Land management objectives and supporting tools**

Objective	Land management tool
Availability of land for (re) development	Expropriation, pre-emption rights, inclusionary zoning regulation, land banking, public land development.
Cost recovery of public works necessary for the development.	Developer contributions, local taxes, urban land adjustment, public land development
Value capturing of the earned increment in land value	Impact fees, local taxes, public land development
Land market efficiency and transparency	Land register, legislation for urban land adjustment.

**Table 2:** **Institutions and their roles in land management in Johannesburg**

Institution	Role in land management
Government	Land acquisition, Land allocation and permitting Land development
Constitutional court	Resolving conflicts arising out of Land Management
Municipality and province	Managing land to meet different demand on land resources in the city, the province also administers the National Housing subsidy scheme.
Johannesburg property Company	Manages city-owned land and property

**Table 3:** **Legislations and their relevance to land management in Harare, Zimbabwe**

Statute	Relevance to land management
Land survey act	provides legislation relating to the survey of land
Urban councils act	Registration of property rights, sale of public land, change of land use reservations,
Derelict land act	provides for instances where the former owner of immovable property cannot be found to sign the power of attorney despite a diligent search
Land acquisition act (Chapter 20:10)	Provisions for compulsory acquisition of land

Urban land administration is a comprehensive system of policies, procedures, and institutional frameworks that deals with the multifaceted process of handling and regulating rights, use, and value of land. This evidently includes determining, recording, and disseminating information about the tenure, value and use of land. Land administration obviously presumes the availability of an information system to enable the determination of the legality of rights to claims on any land. Therefore, without an information system called land registry, no effective, let alone efficient, land administration can be expected. The driver of land registration is the provision of safe and reliable foundation for the acquisition, utilisation and disposal of rights on land (Yusuf, et al., 2009). (Magel and Wehrmann 2002):12 have advocated for the incorporation of “… the norms of (good) urban governance - sustainability, subsidiarity, equity, efficiency, transparency and accountability, civic engagement and citizenship, and security.” Practices including land banking and densification of development have been promising in some countries to ease the burden of disorder and unruliness in the management of urban development. Africa, in particular, lacks public good asset management as argued by (Kaganova 2010): 1 who observes its “…infancy compared with traditional areas like public budgeting or public administration.”

### Some best practices in urban land policies and management

The pace of urbanisation, the rate of economic growth, the availability of land for housing, the increase in land prices and inappropriate strategies for urban planning and land appropriation all directly contribute to this problem. Most governments have tried to implement housing programmes in order to house their urban poor. However, such programmes have recorded minimal success. We examine China and Singapore as offering some of the best practices in urban land policies and management. These countries have managed to unlock the potential embedded in land for sustainability.

#### China

China, as one country in Asia, has distinct land management practices. (Zhang 2012) notes that the country adopted land-banking systems in which there are recognised, hence official, agencies governing local land conversions, preparation, and transactions. This transformation in urban land policy has emanated from the local economic characteristics, pressure from peer cities, provincial governments, policy professionals, and policy-making communities. Over the past years, China has been shifting from a centrally planned to a market oriented economy. Land management issues are critical in China’s transformation. Urban land development planning shapes the landscape and sustainability of rapidly growing cities (Zhang, 2012). The concept of land banking systems was actually borrowed from other cities that were practicing such. Cities such as Dalian, Shanghai, Hangzhou, and Qingdao began to experiment with the land banking system based on the lessons learned from land banking practices in the United States and Hong Kong. In the context of China, such practices had positive results. The new system brings considerable efficiency gains. It enables local governments to regain control over a large share of land conveyance profits that was formerly possessed by illegal land traders and corrupt officials. Moreover, this system enables local governments to enjoy the benefits of land value appreciation driven by China’s rapid industrialization and urbanisation process. In their article, (Ho and Lin 2003) explore China’s land management practices, changes introduced and the rationale behind such transition. Since the early 1980s, China has altered its land use arrangements and introduced new regulations to manage land use changes. In the process, the administrative allocation of land to users has been transformed into a complex hierarchical system of primary and secondary markets for land use rights. The changes in China’s land system were adopted primarily for two reasons: (i) to develop land markets to allocate land more efficiently and (ii) to protect agricultural land. The development of land markets is still at an early stage, that the conversion of land to non-agricultural use continues but at a slower pace, and that illegal land use is pervasive.

#### Singapore

Singapore is one such country that is an exception in market and state failures in land management. Singapore has been able to implement city-planning and urban-management policies that actually benefit the poor, and its housing programme has been successful and admired for producing low-cost, affordable housing on a mass scale (Housing and Development Board 1997). One of the legislation in land policy was the Land Acquisition Ordinance of 1920, which was amended in 1946 and 1955 to give government more powers to acquire more private land for new town development. Due to the extreme scarcity of land in Singapore, the price of land rises very quickly. To allow the authorities to acquire land quickly and cheaply for public-housing programmes, various laws were passed regulating compulsory land acquisition. Compulsory land acquisition has been the most effective way of obtaining land for public development. The Land Acquisition Act also establishes resettlement policies which enable large areas of squatter land to be cleared and for the squatters to be re-housed in low-cost flats. This has given the squatter population a chance to enjoy better housing and living standards (Housing and Development Board 1997). The success of Singapore’s management of urban land resources has been attributed to a number of reasons such as a strong political commitment to public housing; financial commitment which comes in the form of loans and subsidies; legislative support which allows the government to acquire land cheaply and quickly and to exercise legal authority on matters related to public housing development and administration. The success of Singapore’s land management practices has also been linked to the country’s small size and population. Singapore has a comprehensive land information system which meets the needs of all government departments with particular interests in land as well as providing a ‘one stop shop’ for the public. A good land information system base is central to land management practice in obtaining a range of benefits including clear property title, efficient and transparent land transactions, better evaluation of government land policies (Jain, 2008). Having said this, we seek now to provide a profile of the various cities and towns in Africa in relation to how they manage their land with the view of learning how far or near they are to adopting better methodologies.

## Profiling of land management practices in selected African cities

Forthcoming are the urban land management profiles for the following cities in Africa: Nairobi, Abuja, Harare, Kigali, Johannesburg and Addis Ababa. These urban centres have been selected because of their status of urban primacy hence the most populous centres relative to others in their countries. The urban primacy status speaks of their potency in attracting huge population numbers as well as services that demand immense amounts of land for development.

### Johannesburg, South Africa

Johannesburg is South Africa’s economic powerhouse and the most popular metropolitan city in the country. The metropolitan area covers 1,644 km^2^; it is a rapidly growing city, with a population in excess of 3.2 million. The growth rate is 3-4% per annum, resulting from natural increase, as well as in-migration from surrounding areas within and outside the country (Planact, 2007). The rapid growth of Johannesburg has created several problems in terms of services provision and general delivery. Informal settlements, in (and around) the city, lack in basic housing requirements including adequate basic services and amenities; as well as reliable transport. In light of this, the large population of migrants in Johannesburg lack access to the formal urban economy (that is, formal employment, formal housing) which explains the most of these people live in informal settlements, overcrowded rental accommodation and involved in informal employment. In fact, it is estimated that between 150,000 and 220,000 households in Johannesburg live in informal dwellings (*ibid*). The situation in Johanesburg today is, to a large extent, an after effect of the colonial land administration policies in the country.

#### Colonial land management policies

Under the separatist development agenda, later crystallised in the apartheid regime, South Africa was a country exhibiting levels of inequality in wealth and access to services among the highest in the world. A combination of policies and legislation dating from the early 20th century consistently denied Africans vital components of well-being and a secure base in the cities. This gave rise to racial imbalances in the provision of housing, infrastructure and services, which were inherited by post-apartheid local governments. The legacy of apartheid has impacted specifically on the provision of services in Johannesburg in two ways. First, the well-known policy of providing inferior quality services for Africans meant that standards of social and physical infrastructure were intentionally set lower than they were for whites (Beall *et al*. 2000). During the colonial era, South Africa had limited land use rights and provision of infrastructure in black population areas was very poor.

#### Post-colonial land management practices

The post-colonial land management practices in Johannesburg are meant to redress the segregated approaches to land use that was orchestrated by apartheid. The segregated land management approaches saw the development of non white population areas with inadequate or inferior infrastructure and services. Land management practices in the post-apartheid era are directed towards ensuring that well located land is allocated specifically for affordable housing options (Planact 2007). In the city of Johannesburg, land management is done formally or informally. Formal land management is characterised by legal regulated channels that are managed by Government authorities, bureaucratic system often with high costs, and problems in dealing with issues that fall outside existing policies, laws and regulations and it’s a system biased towards owners of land and property, but the use of land can be regulated by the government. On the other hand, informal land management practices are characterised by extra-legal channels that are mainly used by people with an immediate need for land, and who do not have the financial capacity to buy or rent through formal channels. More so, informal networks are often utilized to access land and to address governance within communities as well as instability and lack of regulation. It should be noted that in Johannesburg, formal and informal systems of land management occur at the same time and within the same places (Planact 2007). The main legal framework is the Development Facilitation Act (DFA), which was designed to rapidly release land to achieve national goals of providing housing and services to the poor. However, this Act has been criticised for driving the poor out of the land management activities. In fact, the DFA is often used by developers of higher income housing and commercial development (Planact 2007). There are various actors in the Land management processes in Johanessburg and these include the government, municipality and province, Constitutional court, Johannesburg Property Company.

Key challenges have been identified in as far as land management in Johannesburg is concerned. One of the major challenges is poor institutional coordination (see Table 2). By and large, various government institutions involved in land management do not always work with the same goals in mind, leading to adoption of piecemeal land management approaches. Different agencies in the same municipality dispute over how land is to be used and conflicts between the municipality and the province over how land is to be planned and developed. For the poor, they have very few opinions for residential development, most of which are through the National Housing Subsidy scheme. The Johannesburg Property Company was also found failing to proactively identify land for low cost housing.

### Kigali, Rwanda

Kigali has existed since 1907. It was created by Richard Kandt, in Gakinjiro, very close to the current Kigali City Market, as a German administrative residence. Kigali has been rapidly urbanising (Ilberg, 2008). From 3% in 1970 to 5.6% in 1991, the urbanisation rate has increased to 16.9% in 2002 and 19,3 concurrently. The urbanisation processes have happened in a rapid and uncoordinated manner that the social services, employment and basic infrastructure are lagging behind (Manirakiza, 2012). Urbanisation has not been matched with a corresponding increase in provision of services and infrastructures facilities to the majority of the urban dwellers like transportation, housing, education, health, sewage and sanitation infrastructures. Over 70% of Kigali city is occupied by informal settlements; most of which are found in poor neighbourhoods. The 2002 general census revealed that around 58% live in spontaneous settlements and 22.8% in isolated ones. While 43.1% live in their own houses, 47.6% are tenants. There are inadequate urban services and few amenities. Only 20% have water in their households, 45% buy water from vendors while 28.4% fetch water from boreholes and natural springs. The urban population growth has intensified informal settlements on land that is considered inappropriate for development like steep hills or valleys with almost no amenities or basic infrastructure available (Ilberg, 2008). This has exerted further pressures on the already inadequate infrastructure and stands as an obstacle to the urban development, planning and upgrading.

#### Colonial land management policies

Colonisation introduced new elements in the Rwandan society, both exogenous and dominating, that were going to bring in changes and distortions in domestic social balances (Österberg et al. 2006). Belgian colonization introduced also the written law appearing in the “codes and laws of Rwanda”, particularly in order to guarantee land tenure security for settlers and other foreigners wishing to invest in land in Rwanda. The Belgian colonial administration established the 1885 decree concerning land use, the provision, of which introduced the duality of systems in the country’s land tenure system**.** The 1926 reform divided the country into chieftainships and abolished the system by which a chief could own several land properties in different parts of the country, which characterised his importance in the country’s hierarchy. And yet this form of the management of the country had been a factor of national unity and cohesion. The abolition of these traditional structures for the purpose of exercising better control of the country and get colonial orders accepted caused a lot of disturbances to the Rwandan society. Owing to the high population density and the need to exploit new areas, the colonial administration introduced the system of grouped homesteads called “paysannats”, which was similar to the traditional system of “Gukeba”. This practice was introduced after the abolition of the “Ubuhake” system and the distribution of cattle in grazing areas [Ibikingi]. It promoted the extension of cultivated land to the detriment of livestock. This development gave rise to conflicts; both latent and real. Thus, large sections of the population among cattle breeders migrated to Umutara, Uganda and Congo. Between 1952 and 1954, King Mutara III Rudahigwa abolished the system of “Ubukonde” and decreed that all the “Abakonde” would henceforth share their land property with their tenants, known as” Abagererwa”. From 1959 onwards, the land tenure system became a factor of real conflict among the population. It was during this period that, with the eruption of the political crisis, the first ever wave of refugees went into exile, leaving behind both their landed and real estate properties.

### Post-colonial land management practices

After the independence, the situation has not changed much. 90% of the country’s arable land is still governed by customary law. The written land law still applies to a small number of persons and religious congregations. It applies more often in urban areas and business communities. During these periods, the government gave an important role to the “communes” in the administration of land. Through the ‘Loi Communale’ of 23/1/63, the protection of rights relating to registered land under the customary law became the responsibility of the commune (see Niyonsenga, 2013; Rwanda Governance Advisory Council, 2011; Umoh, 2012). However, the provisions of this law were virtually nullified by Decree No. 09/76 concerning the purchase and sale of customary land rights or land use rights. While at the beginning of the 60’s the Government banked on abolishing the system of “Ibikingi” to put them under the authority of the “communes”. On recovering the land abandoned by the 1959 refugees to acquire new agricultural land, the 1970–1980 decade was characterised by intensive migration from the already densely populated regions of Gikongoro, Ruhengeri, Gisenyi and Kibuye to the semi-arid savannas of the East [Umutara, Kibungo and Bugesera] in search for vacant land. In 1976, decree No. 09/76 of 04/03/76 concerning the purchase and sale of land customary rights, or the right of soil use, authorized individuals to purchase and sell customary land after application to the competent authorities, and subject to retaining at least 2ha of land. The buyer was also to justify that he did not have land property equal to at least 2ha. Ever since, the Government recognized only the right of ownership based on land registration and became, therefore, the eminent land owner. At the beginning of the 80s, there were no more new lands, and problems began to emerge bluntly; reduction of soil fertility and of the size of land for cultivation, family conflicts stemming from land ownership and food shortages. From 2ha in 1960, the average area of a family’s cultivation plot was reduced to 1.2ha in 1984. Since the beginning of the 90s, the country found itself in a land-related deadlock. Problems included insufficient agricultural production, increasing population pressure on natural resources, growing number of landless peasants, and conflict between agriculture, livestock and natural reserves.

Rwanda appears to be on good way with regard to land management (see Niyonsenga, 2013; Rwanda Governance Advisory Council, 2011; Umoh, 2012). Land registration and management policies have been elaborated and completed. Land tenure is legally ensured for every owner. Although efforts have been made in this regard, urbanisation issues based on land issues are still alarming because of the rapid urbanisation process (Manirakiza 2012). In Kigali, GIS has been incorporated in urban land management to increase land tax collection revenue (Österberg et al. 2006). Kigali City Council (KCC) Cadastral Information System is linked to a land tax revenue billing system could pay back the development investments in a relatively short time. A new land policy draft was finished in January 2004, and the land law for its application was published in September 2005. The Land Policy calls for rational use and sound management of national land resources and be based on master plans. The Policy also provides development of land use plans based on suitability of the areas/ lands/swamps thus distinguishing the different categories of land and their purpose. The current urban land market in Kigali is not delivering sufficient quality of land for the poor and increasing size of migrants. Therefore, the land and housing markets must be regulated and urban planning policies are to be initiated in their favour rather than pushing them in slums and in urban fringes; otherwise they will continue to plague the sustainable urban development through constant urban sprawl (Manirakiza 2012). Tenure insecurity for Kigali’s residents was and still is high. Most land in Rwanda is individual land holdings, and evicting people to create space for new development or private investment is common in Kigali. Corruption is also a critical challenge in land management in Kigali. Corruption is perceived as one of the most important issues that causes the delays in delivering and issuing land documents.

### Nairobi, Kenya

Nairobi, like many cities in Africa is experiencing rapid population growth. The population of Nairobi grew from 8,000 in 1901 to 118,579 in 1948 (Rakodi 1997). By 1962, the city had a population of 343,500 people, although some of this could be attributed to extension of the city’s boundaries. Between the 1948 and 1962 censuses, the population grew at an average rate of 5.9 per cent per annum, compared with 7.6 per cent in the previous 12-year period. Taking the 1999 census figures as a baseline, it is projected that the city’s population by the next census in 2009 will be about 3.1 million, and 3.8 million by 2015 (CBS 2001). Rapid urbanisation in Nairobi will exert more pressure on the available resources.

#### Colonial land management policies

The first Land regulations in Kenya were passed in 1891. These regulations formed the framework for land adminstration. The rules provide 21year leases. There were two land laws that defined the course of land policy in Kenya and these were (1) Crown Land Ordinance of 1902, which empowered Commissioner of Lands to grant leases on crown land. This ordinance however, did not deal with the question of the extent of indigenous land rights. The law was then repealed and gave birth to the Crown Lands Ordinance Act of 1915. African were only restricted within native reserves and could not own land outside these reserves. In 1933, the Carter Land Commission was set up by the colonial government to investigate land grievances and to assess land needs of the indigenous population (Lamba (2005).

#### Post-colonial land management

Land tenure systems in the slum settlements of Nairobi represent perhaps the most awesome extralegal land administration system in Kenya. De Soto (2000) states that extralegal systems are adapted when ‘the cost of obeying the law outweighs the benefits’ De Soto adds that”… the migrants become extralegal to survive: they stepped outside the law because they were not being allowed inside”. Land administration processes in Kenya have been described as inefficient, bureaucratic, corrupt, expensive and colonial (GOK Government of Kenya 2009). These characteristics of the land administration system are unaffordable and inaccessible to the poor and thus serve to exclude them from formal property ownership resulting in informal property ownership systems and informal development in the urban areas. After being locked out of the legal system by the factors narrated above, the informal developers may device their own extra legal systems of accessing, securing and disposing of their rights in land (Omwoma, 2013).

### Addis Ababa, Ethiopia

Addis Ababa was founded at the end of 19th century by emperor Menelik ll. The city is located at the geographical centre of Ethiopia (8°55’–9°05′N and 38°40’–38°50’E). The municipal boundary is estimated to enclose a total area of 540 km^2^, of which 18.2 km^2^ are considered to be rural. Currently, Addis Ababa is inhabited by a population of more than 3.2 million, which accounts more than 4% of the total population of Ethiopia. The city is characterised by rapid growth and subsequent planning difficulties that have arisen from the past urban planning and management practices. Addis Ababa is one of the fastest growing African cities where provision of housing, infrastructure and services is no more in pace with the growth of the city. Even though there have been various interventions to harmonize the growth of the city with the overall qualities of life, inadequate housing and infrastructure in the city have been major problems since its foundation (Mahiteme, 2009).

Land delivery systems in Ethiopia have undergone different land tenure systems. This has largely been a reflection of the prevailing land policy and land holding tenure systems of the country under different governance regimes. Notable examples include the free hold land tenure system (pre-1975), public controlled permit system (1975–1992) and public lease hold system (1993 up to date). Besides, these there are also customary and informal land holding systems, which are commonly known in Ethiopia and other developing countries (Gondo 2009). The major formal land delivery system for residential housing and investment in Addis Ababa and other big cities is through the lease mechanism. But in some smaller towns it is on a rental bases. Land is a public property and an individual can enjoy only the use right of land under his/her possession. Thus, the means to acquire legally (formally) a plot of land for housing development, and investment purpose is dependent on the efficiency of lease policy application. Lease Proclamation No 172/2002, is the current active law regarding land provision, and indicates different ways how one can acquire a piece of land. These include auction, negotiation, lottery system and through an award system (ibid). In 1994, a new Ethiopian constitution was promulgated. The constitution retains state ownership of the land. Article 40; sub-section 3 of the constitution states that land “is exclusively vested in the state and in the peoples of Ethiopia. It further stipulates that ‘land is a common property of the nations, Nationalities and Peoples of Ethiopia and shall not be subject to sale or other means of exchange.”

While every Ethiopian citizen has the right to own private property as provided for in article 40; subsection 1 of the Constitution, the Constitution does not provide for private ownership of land. The land tenure system for urban areas is comprehensively dealt with by the Urban Lands Lease-holding Proclamation No. 172/2002. Land is allocated through the leasing system. While the leaseholder of urban land is free to dispose off part or all of the interest by sale or other means of exchange, the lessee of public land is prohibited by law to sell the land or enter into any contract that binds the land. The policy allows that the government can retain land needed for public interest and individual holdings for better development activities by paying compensation to owners for the properties located on such pieces of land. Ethiopia reflects a very different dimension is as far as land management is concerned.

The important dimensions of the land policy include, allocating land in a sustainable way through tender, negotiation and permit, ensuring the equitable distribution of land to both the rich and the poor, facilitating mechanisms by which low income groups are allocated adequate land at reasonable cost, bringing economical uses of land for intended development works (Urban Development Policy 2005) stabilising the price of land and marketing of real property. Urban Land Policy gives priority to land allocated (in order of priority) to saving houses, social services, industry, micro and small business institutions, residential houses, commercial organizations and recreational centres. The relationship between land and poverty is clearly articulated in Ethiopia’s Plan for Accelerated and Sustained Development to End Poverty (PASDEP) policy document (2005/06- 2009/10). Improved access to land, infrastructure and facilities is one of the four pillars of PASDEP that is aimed at eradicating poverty (Gondo 2009). In Addis Ababa, the central and local governments or municipalities are critical actors in Land Management processes. These institutions are responsible for the promulgation of Legislation and policies which regulate the practice of land management (acquisition, allocation, development, transfer, land taxation etc.). Central governments have a key role in defining land related policies whilst most land management related operations take place at the municipal level. The Land Administration Agency in Addis Ababa city Government is responsible for providing serviced land in efficient way for housing development.

The land delivery systems in Addis Ababa is also characterised by informal actors such as residents of the informal settlements, land brokers, land speculators, local farmers and state agents. Local residents, land brokers, gatekeepers, speculators, local officials and local labourers are the major actors in land delivery systems in Addis Ababa. Local officials include both local politicians and professionals. A study made in 2003 mentioned corruption as one of the major causes of illegal land occupation and transaction (Mahiteme 2009). Another study in 1999 had already revealed that lack of clear rules and regulation, a weak institutional capacity and corruption were the primary causes for inefficient urban land use and uncontrolled land occupation. These claims were also confirmed by most of the experts in the Land Administration and Development Authority. Therefore, local officials are identified as key actors in the informal land subdivision and transaction (Mahiteme 2009). Local residents are either the original landowners or squatters who own plots through informal subdivision. They are usually perceived as marginal actors once they have sold their land or secured their own plot. They also play a key role as information centres for the newcomers who want to buy land in some areas. They also act as sub-brokers by leading the new buyers to the main land brokers. The main activity of land brokers is to bring buyers and sellers together. Governments around the world pursue urban land policy objectives and they rely on a vast range of policy tools and institutions to achieve them. In Addis Ababa, there are various tools for achieving the objectives of urban land policies and these include master plans, zoning, subdivision regulations, building codes, and other public policies to shape development. These regulations are normally adopted to help protect the urban and natural environment, gear infrastructure investments with development, and maintain and enhance property values.

Private sector involvement in policymaking in general, including land and land management policy, is minimal in Ethiopia. Public-private partnership is virtually absent. Before the nationalization of urban land in 1975, both urban and rural lands could be sold, rented, leased, inherited or transferred as a gift. Proclamation 47/1975, which introduced the monopoly of land ownership by the state, abolished private ownership of land and banned any form of transaction in land. The state took over total responsibility for land provision, development, and control. Accordingly, urban land management became a highly centralized operation under the then Ministry of Housing and Urban Development (MHUD) that had oversight over the implementation of the policy through its regional branches and urban dwellers associations established at the city and neighbourhood levels (Yusuf, et al. 2009). Major challenges in land management in Ethiopian cities arise because of rising urban informality. The major cause of such informality has been the challenges or flaws associated with the land administration process among other factors that relate to historical land tenure systems and urbanisation. The demise of the formal land supply has subsequently seen the emergence of opportunistic and informal of land supply tendencies. Many households have turned to the informal land markets to compensate for the deficits of the formal land supply market. Rent seeking behaviour has also been on the rise in the private market, with the bulk of private land suppliers resorting to speculative behaviour responsible hiking the value of land parcels. The deteriorating land supply situation in Addis Ababa has seen most people resorting to informal land delivery systems.

### Abuja, Nigeria

Abuja the new capital of Nigeria came into existence by virtue of the Federal Capital Territory Act, of 1976. The Territory covers a total land area of approximately 8,000 square kilometres, while the City proper is to cover a total land area of 250 square kilometres (JIBRIL 2006). Abuja was made the federal capital territory of Nigeria after the population boom in the former capital Lagos necessitated the move of the capital to a less populated environment. Abuja has always been known to be a well planned and tranquil city, but in recent times the city is beginning to witness a huge population growth stemming from the migration of people to the city and this has resulted to the expansion of settlements outside the city limits and even the building of residential blocks in areas meant to be left fallow according to the city’s original master plan. The strain of the population boom on the nation's capital has resulted to some problems like sanitation and traffic congestion. During the close of the day's work, it is a norm to see stranded and exhausted citizens waiting at major junctions to catch the popular Bus Rapid Transit (BRT) buses or the small green and white buses back to their homes far away from the city center. This rush wil mean more traffic congestion, a constant hum of blaring horns and a chaotic atmosphere for the people of the city.

#### Colonial land management practices

The Colonial Nigeria was divided into colonies and protectorates where multiplicity of land tenure systems existed. The arrival of Europeans in Southern Nigeria in the later part of 19th drastically changed the land holding system. As soon as the European traders, who were used to freehold, began to acquire land parcels in Lagos colony, they did so with the concept that the transactions conferred on them absolute ownership and the right of alienation. However the Northern Protectorate was saved the experience of Southern Nigeria. Lord Lugard, who occupied Northern Nigeria at the turn of 19th century, used the “tools” he found locally for the administration of land holdings. The Emirs who exercised “proprietorial” rights were appointed or re-appointed and given “letters of appointment” which transferred their feudal pattern of land holdings to the Crown. The Native Rights Proclamation of 1910 nationalised all land and placed it under the control and administration of the Governor in the interest of the indigenous population. In 1914, Lord Luggard amalgamated the Southern and Northern Protectorates into one centralised Nigeria ruled from Lagos, with each region retaining its land tenure system. In 1954 under regionalisation scheme, three regions emerged with the Northern Protectorate becoming the Northern Region, and the Southern Protectorate divided into West and Eastern Regions. The Regions were subsequently divided into States starting with 12 State in 1968 till the current 36 States and Federal Capital City, Abuja. Each state inherited the land tenure system from the Region it was created (Atilola 2010).

### Post-colonial land management practices

The Land Use Act of 1978 is the principal law guiding land acquisition, resettlement, and its allocation to all eligible Nigerians; private, government agencies and nongovernmental organisations. It provides for the Government to hold land in trust for the use and common benefit of all Nigerians, for the realisation of equity, fairness and justice in the control and management of land, resettlement and compensation purposes. This ideal cannot be achieved without adequate and efficient land administration tools. One of those tools of course is a reliable and up-to-date land records. Institutions involved include the Federal Capital Development Authority (FCDA) and the Federal Capital Territory Administration (FCTA), which are responsible for maintaining manual record keeping right from inception (about 29 years. This system of land registry has many problems such as Multiple allocations of plots, Land Use Abuses, Encroachments, Inefficient system of Revenue Generation, Proliferation of Unplanned/Squatter Settlements, The use of obsolete Survey Equipment, Rampant subdivisions and redesign of plots and Extensions beyond the Federal Capital City Master Plan limits (Jibril, 2006). This has seen the Government comprising the land registry system in 2003. The computerized system has many capabilities. It can give information on landed properties, cadastral maps, land records, land use. The information products can be used for decision support in land allocations and all kinds of land related matters like detecting, documenting and resolving cases of multiple allocations of plots, encroachments on corridors of roads, water- and sewer trunk lines as well as land use mismatches (Jibril 2006).

### Harare, Zimbabwe

Harare is the capital city of Zimbabwe. Since its birth in 1890 as Salisbury, it has seen some tremendous growth. Under colonial legislation and policy, there were three basic areas in cities: the part for Europeans and all whites only; places for Asians and coloureds; and places for Africans. This kind of city was racialist and exclusive (Chirisa 2013). Harare’s growth rates reached over 5% per annum throughout the 1980s (Zinyama et al. 1993). This strained the capacities of both central and local spheres of government to provide housing and basic urban services for the urban poor.

#### Colonial land management practices

Starting from the colonial period and continuing into the post‒independence period, transactions in land and other forms of property in Zimbabwe’s urban areas were, in general, undertaken through formal land markets. A common feature of the country’s main urban areas is that they were established during the colonial period. By and large and for racial reasons, the cities and towns were located in the midst of privately owned, large-scale commercial farmland. Regulated by the powerful Regional Town and Country Planning (RTCP) Act and the Urban Councils Act (UCA), the development of Zimbabwe’s urban areas was guided by formal town planning, surveying and land registration processes. Guided by the same town planning and development standards, the post‒independence government did not permit the development of slums or informal settlements in various parts of towns and cities (Zinyama et al. 1993). Essentially, the new government strongly opposed the development of unauthorised/informal settlements within urban areas. Consequently, squatter settlements were generally destroyed wherever they appeared. Urban development in Zimbabwe, in the pre‒1980 period, exhibited a false efficiency in terms of land delivery, since demand by black Zimbabweans was suppressed through legal instruments (e.g. the pass laws) and in terms of the development model that was applied. However, the removal of such restrictions to black land ownership and permanent urban residency after independence saw a surge in demand that outstripped land supply by the early 1990s (Marongwe et al. 2011).

#### Post-colonial land management practices

The legal framework governing the operation of urban land markets includes Acts of Parliament, statutory instruments, government policy documents and Ministerial directives. The principal legislation relevant to the operation of urban land markets includes the Regional Town and Country Planning Act and the Urban Councils Act (for land delivery), the Land Survey Act (for title survey), and the Deeds Registry Act and the Derelict Lands Act (for the registration of property rights) (refer to Table 3). In terms of government policies and directives, the land policy, the national housing policy, the policy on incremental and parallel development and other ministerial directives affecting access to land by different actors are key instruments that govern urban land markets.

There are various actors involved in land management processes in Zimbabwe. These include the Ministry responsible for Lands, which is the principal land acquisition authority. It manages the Office of the Surveyor-General, which runs key land information systems and administers rural state land. The Ministry of Justice, which is the custodian of the Deeds Registry has functions which include administration of the property conveyance system. The City of Harare which is responsible for land allocation and administration as well as land use planning. Another important player in the land management processes in Zimbabwe is the private sector, which includes land development companies such as pension funds and building societies. The year 2000 saw the birth of a bitterly disputed Fast-Track Land Reform Programme (FTLRP), which resulted in the transfer of land mostly from white commercial farmers to the black majority. The collapse of Zimbabwe’s land management and administration system could also be traced to this era. The government of Zimbabwe started implementing the Fast-Track Resettlement Programme in July 2000. The ultimate objective of the programme was to accelerate both land acquisition and land redistribution. Fast track resettlement programme is officially viewed as a component of the overall National Land Reform Programme (Marongwe 2003). The land management processes in Harare has several challenges. There are unclear responsibilities on aspects of land management between the ministry responsible for lands and the City of Harare. The processes of land acquisition are cumbersome and lengthy and it clogs land delivery processes (Government of Zimbabwe, 2009).

### Emerging issues: sifting between expectations and realities

The embryonic challenges among African cities in as far as land management is concerned include poor governance frameworks, which results in poor management of land, lack of appropriate land management systems to deal with increasing shortages of serviced land and well sited land, widespread informal land delivery systems, highly centralised land agencies, incomplete land records and poor public land management. In addition, increasing housing informality, coupled by uncollected refuse and the proliferation of the informal sector activities sum-up the limitations of sustainable land governance options. Because of these challenges, the urban populace is often in limbo with reverence to what they can do for themselves as they are caught in between forces of market and state failures in efficient land delivery.

As for example, from the case study of Johannesburg, large migrants in Johannesburg lack access to the formal urban economy (that is formal employment, formal housing) which explains that the most of these people live in informal settlements, overcrowded rental accommodation and involved in informal employment. As a result, key challenges have been identified in as far as land management in Johannesburg is concerned. One of the major challenges is poor institutional coordination. This critical problem does not only apply to Johannesburg only but affects also other African cities of which Harare and Addis Ababa are not an exception. People are accessing housing through a variety of established channels such as cooperatives, home ownership schemes, ‘Pay for your own house’ schemes and private sector investment programs. Furthermore, the failure to provide adequate housing has exacerbated self-provisioning of residential sites with infrastructural inefficiencies and deficiencies and problems of insecurity (physical and health), arising from overloading, in-serviceability and high cost service provision demands. This confusion reflects that weaknesses in key institutions are an essential productivity snare and a crucial aspect of informality. The chief expression of urban informality is in the housing sector hence slum formation. This phenomenon reflects that Africa in particularity lacks public good asset (land) management.

The scenario has also been fuelled by extensions of city boundaries by various African Local authorities. The extensions of urban boundaries for residential development come with many challenges than solutions. Initially, it goes against contemporary global concept of smart growth, which encourages vertical development rather horizontal. Further concerns are witnessed from the strain inserted on the available service especially water. Contemporary urban residents have developed a different mindset and are no longer concerned whether the land being allocated to them is serviced or not. Their minds are now obsessed with possession of land and nothing else. However looking on land management in Africa there are quite a number of factors that often hamper sustainable urban land management. Currently, most African cities lack a comprehensive land policy and one wonders how a country can operate without a land policy. Furthermore, over-centralisation of land administration and urban planning also hamper sustainable land management in Africa. At the end of the day, this factor has given birth to corruption that has of late been a cancer in African cities. Moreover, politicians who seem to have the bragging rights to parcel land willy-nilly have usurped urban planning. The reality on the ground is confusing as planning has been rendered a free for all especially in Zimbabwe. Council officials, councillors and workers, have taken up town planning roles by storm in such a way that subdivisions of open spaces have become the order of the day.

In summary, one can note that there is an increasing poor performance public land management in providing land for housing, registering titles and land transfers, regulating access to and use of urban land as well as providing basic infrastructure services. Parallel development which follows the ‘built, occupy and service later approach’ has seen African cities adopting it at the detriment of public health concerns and sustainable land management. Although it is an initiative that makes housing affordable, one must note that affordability comes in many forms. Actually this initiative is more expensive and further exacerbates housing shortage. As a result, one can argue that the notion of housing should be considered in the context of settlement (land) management. Therefore, people must not advocate for housing alone but rather look for ways and means of creating and distributing housing services to meet individual needs and satisfy the goals of the larger society. That is from the use of housing, flow a variety of services to the household-satisfaction, status, privacy, security and equity as well as shelter and services to the original builders and a host of other agents.

#### Which strategies work and how?

Since many government interventions are inefficient and lead to sub – optimal distributions of land resources, some policy experts argue that the best way to “manage” land use and development patterns are to rely on market forces. On the other hand, without planning and regulations, land markets are likely to generate enormous external costs and fail to produce public spaces. In fact, without government intervention critical public facilities such as parks, open spaces, and major infrastructure and urban services, which the private sector cannot profitably produce and sell, will not be provided. Thus, the solution to ineffective and counterproductive urban land policies is not to do away with governments interventions and policy initiatives, but to find the proper balance between the public and private sector regarding urban land development and management (Yirsaw, 2010). The six cities use different strategies and approaches to land management and such approaches have their own strengths and drawbacks. In Kigali, for example, decentralised land management structures have proved to be effective since leaders are accessible and the land registration processes are quickest. Unlike, in Harare, where there is too much centralisation of land management, characterised by bureaucracy, corruption and lengthy land acquisition, land allocation and land registration processes. Formal public land management strategies have been ineffective in involving the urban poor in land markets, and this has seen informal land delivery systems giving the poor an opportunity to participate in the governance of urban land markets.

The governments in all the reviewed cities should amend land acts and policies to include alternative and innovate tenure systems that can carter for the interest of the poor in urban areas and make documentation of rights to land easy, cheap and simple to understand. As a stopgap, the rights of tenure of those occupying public land in the informal settlements should be respected by invoking the bill of rights, which gives them a right to shelter. Under conditions of rapid urbanisation, competition for secure and serviced land increases. This places pressure on existing tenure systems and requires governments to formulate policies, which encourage efficient land use and improve accessibility to land, without sidelining the urban poor. The central policy issue, hence, becomes what forms of land tenure best achieve these objectives of efficiency and equity (Yusuf *et al*., 2009).

#### Discussion, policy implications and recommendations

The discussion in this paper is not necessarily exhaustive but it potentially provides scope for debate on the land management issues in Africa. The current relatively incoherent economic policy framework needs to be dramatically adjusted to address more effectively the key land management problems affecting Africa. It is in this context that there is need for rethinking of the ideology underpinning the policies being implemented. It is also important to recognise that even though economic mismanagement within government exists, Africa like many other continents remains a post-colonial continent unfairly inserted within global capitalism. Therefore, critical questions can be asked on whether domesticability of exotic strategies can add value to the status of land management in Africa. Domesticability is possible but needs a closer analysis before implementation takes place. What works in one country cannot work in another due to various constraints. This leads one to the global-rhetoric argument where translation of the said exotic or global polices to local countries receptivity is critical. However, one can also talk of argue from a standpoint that the norm of domesticating exotic strategy is not sustainable. This is because Africa itself knows her own problem and can as well design its strategies based on experience rather than inspired guesswork or assumptions. Therefore, it is imperative that one analyse the situation at hand before fully domesticating certain strategies.

The recommendations for sustainable land management in Africa are derived from the challenges the continent is facing good governance on the part of local authorities of various cities. As the norms of (good) urban governance includes sustainability, subsidiarity, equity, efficiency, transparency and accountability, civic engagement and citizenship, and security, its practice will see African cities curbing poor land management practices. Good governance on a bigger scale reduces corruption-a cancer that has largely affected various African cities. Planners must also resist from being reactive but must be proactive in the sense that reacting to problems reduces efficiency as one has to compromise in some instances. In addition, planning also needs to move from being rigid to flexibility to cater for the ever-changing African economy. This is because the dynamism in land management assumes the active participation of the citizens including the importance of future generations in the plans. The current bottlenecks to low cost housing supply derive from a rigid mind-set on the part of government and local councils that they are the only ones capable of delivering low cost housing. Experiences elsewhere have shown that a regulated private sector (mostly in partnership with the local councils) can deliver low cost housing at a faster and a more efficient pace than governments. Currently housing investments in most African countries are focused on servicing the upper middle class and the elites given the cash stringent economies Africa finds itself. As for example, in Zimbabwe, the Reserve Bank of Zimbabwe led Home-Link was potentially one of the credible solutions to the housing problems in Zimbabwe but it did not receive adequate support for a variety of reasons. There is need to establish a similar mechanism/fund for low cost housing elsewhere. The major reason being that from the use of housing, flow a variety of services to the household-satisfaction, status, privacy, security and equity as well as shelter and services to the original builders and a host of other agents.

Flexible planning, public-private partnership, low-cost technologies and appropriate standards are key strategies for sustainability. Effective land administration posits the availability of an information system to enable the determination of the legality of rights to claims on any land. It is therefore imperative that local authorities exercise effective land administration. An information system like GIS if used is another critical tool under land administration especially in creating a land register. Without land registry, no effective, let alone efficient, land administration can be expected (Yusuf et al. 2009). Land registration is largely carried out with the prime objective of providing safe and reliable foundation for the acquisition, utilization and disposal of rights on land. The practices of land banking and densification of development can also go a long way in solving land management problems in African cities. These strategies must be supported by a firm policy for land management to ensure everything is done under laid down procedures. Thus, according to (Yirsaw 2010), the solution to ineffective and counterproductive urban land policies is not to do away with governments interventions and policy initiatives, but to find the proper balance between the public and private sector regarding urban land development and management. The following specific proposals can enhance better land management:

- Enhance understanding of and compliance with laws related to land rights.
- Adhere to expropriation related procedures as provided by the Law.
- Enhance mechanisms to improve Land service delivery.
- Pursue policies to mitigate the underlying causes of land disputes.
- Strengthen mechanisms to combat small-scale land related corruption at lower at lower administration levels (village, cell and sector).

The idea is that the proposed recommendations will provide an opportunity to city authorities, civil society and other stakeholders to work together to ensure that services in land management and administration are better delivered to the citizens. Overall, Rwanda’s land tenure system requires comprehensive reforms, from the elaboration of a national land policy to the establishment of a land law and land code, which will guide the judicious use and management of the land resource.

### Conclusion

Effective urban land control is crucial to tackling growing land use problems such as slum formation, rising costs of land, accessibility to urban land for land housing, incompatible use, flooding, overcrowding and congestion among others for the purpose of achieving sustainable city development and ensure the safety and health of the people. There is increasing poor performance of formal (public) land management in providing land for housing, registering titles and land transfers, regulating access to and use of urban land as well as providing basic infrastructure services. The deficits of the public sector have, however, been largely compensated by the increasing importance of an informal subsystem in housing land development (Kombe and Kreibich 2001). The Emerging challenges in land management among African cities include poor management of land and poor governance frameworks, lack of appropriate land administration systems to deal with increasing shortages of serviced land and well-located land, widespread informal land delivery systems, highly centralised land agencies, incomplete land records and poor public land management. The obtaining trends on the ground indicate deterioration in the tenure status and access to land for the poor (UNCHS 2001). Public and private formal land delivery systems have failed to cope with the needs of the urban poor. One of the notable deficiencies of the formal land delivery system in urban areas of the developing world has been (as argued earlier on), the emergency and proliferation of informal elements. Informality has manifested itself at various scales of the land management system, including land acquisition, land delivery process, land titling among others. Among a host of other symptoms of such deficiencies have been the proliferation of illegal settlements and the unprecedented rise of legal disputes associated with land management issues.
